# High-throughput sequencing technology facilitates the discovery of novel biomarkers for antiphospholipid syndrome

**DOI:** 10.3389/fimmu.2023.1128245

**Published:** 2023-05-19

**Authors:** Qi Liu, Shuo Yang, Yuan Tan, Liyan Cui

**Affiliations:** ^1^ Department of Clinical Laboratory, Peking University Third Hospital, Beijing, China; ^2^ Core Unit of National Clinical Research Center for Laboratory Medicine, Peking University Third Hospital, Beijing, China; ^3^ Institute of Medical Technology, Peking University Health Science Center, Beijing, China

**Keywords:** antiphospholipid syndrome, next-generation sequencing, biomarkers, diagnosis, prognosis

## Abstract

Antiphospholipid syndrome (APS) is characterized by arterial and venous thrombosis and/or morbid pregnancy, accompanied by persistent antiphospholipid antibody (aPL) positivity. However, due to the complex pathogenesis of APS and the large individual differences in the expression of aPL profiles of patients, the problem of APS diagnosis, prognosis judgment, and risk assessment may not be solved only from the antibody level. It is necessary to use new technologies and multiple dimensions to explore novel APS biomarkers. The application of next-generation sequencing (NGS) technology in diseases with a high incidence of somatic mutations, such as genetic diseases and tumors, has been very mature. Thus, we try to know the research and application progress of APS by NGS technology from genome, transcriptome, epigenome and other aspects. This review will describe the related research of NGS technology in APS and provide more reference for the deep understanding of APS-related screening markers and disease pathogenesis.

## 1 Introduction

Antiphospholipid syndrome (APS), previously termed as Hughes syndrome, is a chronic autoimmune disease characterized with venous or arterial vascular thrombosis and pregnancy morbidity (1). Reliable epidemiological studies on antiphospholipid antibody (aPL) and APS are still largely lacking. Limited statistics just show that the annual incidence of APS is about 2-5 cases per 100,000, the prevalence is about 40-50 cases per 100,000 and the 10-year mortality rate is approximately 10% (2–4). Currently, the key laboratory tests for clinical diagnosis of APS are three standard aPLs: anti-cardiolipin antibodies (aCL), anti-β2 glycoprotein I antibody (aβ2GPI) and lupus anticoagulant (LA). APS patients often have multiple organs-systems injury and develop genetical predisposition, associated with the persistent presence of serum aPL (5). However, there are many problems in the practical application of the above three standard aPLs. APS patients have diverse clinical manifestations, even some atypical clinical manifestations are also frequently seen in APS patients. Numerous scholars have proposed non-standard clinical presentations and called for the refinement of new criteria to classify patients with increasingly recognized non-standard neurological presentations (6, 7). The expression of aPLs is relatively complex, suggesting that there may be non-aPL related clinical manifestations (8). Some reserchers have proposed a new type of APS: seronegative APS (SN-APS) (9). In some cases, patients may present with clinical features of APS but with temporary positive or persistently negative titers of aPL, meaning that these patients with high clinical suspicion of APS but cannot be diagnosed as APS because they do not meet the laboratory test diagnosis criteria, which may cause a certain impact on the treatment and prognosis of patients. Moreover, Gilberto et al. have proposed “non-criteria” APS subgroups, such as aPL carriers and patients with “non-criteria” manifestations and positivity for “non-criteria” aPL (10). Although many studies have explored the clinical value of non-standard antiphospholipid antibodies, which may provide hints on some conditions (11, 12), the new APS classification criteria process is well under way and some initiatives are being proposed (13–15). It is reported the committee recommend the removal of tests for anti-domain-I antibodies (aDI), anti-phosphatidylserine-prothrombin antibodies (aPS/PT), aCL IgA, aβ2GPI IgA, anti-prothrombin antibodies, resulting from their limited commercial availability, additional studies which are required to define feasibility, clinical relevance and standardization. However, the positive rate of the same type of antibody varies greatly among different populations in many studies. In addition, aPL is also positively expressed in other autoimmune diseases, infections, tumors, vaccinations, and even healthy individuals (16). 30-50% of patients with systemic lupus erythematosus (SLE) are reported to be positive for at least one aPL test (17), meaning the specificity and sensitivity of aPLs may not meet the needs of clinical diagnosis and treatment. These problems together lead to the heterogeneity and complexity in the diagnosis and prognosis of APS patients, and bring a double burden to patients mentally and economically. Therefore, early diagnosis, early treatment and intervention, drug action prediction and prognosis of APS are important clinical problems to be solved urgently. Reserches with larger number of samples and longer follow-up time are still needed to judge the application value of non-standard antibodies in future.

In recent years, the continuous development and innovation of NGS (next-generation sequencing) technology has greatly promoted the integration and development of all fields of genome research (18). NGS technology mainly obtains DNA sequence data by library construction, cross-linking amplification between library and vector, synthesis and sequencing on top of vector (19). At present, this technology is gradually moving from the research field to clinical practice, and the main technology categories include: whole genome sequencing (WGS), whole exome sequencing (WES) and target region sequencing (TRS), etc (20). As an “open system” for exploring new information and identifying existing information, this technology have higher sequencing capacity, ability to multiplex samples, and lower sequencing cost if batched. The massive sequencing capacity of NGS permits its broad research and clinical applications in different fields, such as diagnosis of diseases (21), microbiome determanition (22), detection of gene mutations (23) and so on. By using new technologies such as NGS and omics, researchers have explored the pathogenesis, clinical phenotype and biomarkers of APS in different directions. Meanwhile, multiple international multidisciplinary research networks and cooperative projects have been established (24) to further promote the in-depth study of the core mechanism of APS pathogenesis, and promote the mining and verification of novel biomarkers. It provides a new way to improve the existing problems of early diagnosis and prognosis of APS.

## 2 Application of high-throughput sequencing technology in the pathogenesis of APS

The pathogenesis of APS is multifaceted, and a growing body of evidence supports that the development of APS is caused by aPL mediated, interactions between inflammatory and thrombotic mechanisms centered on disruption of coagulation and fibrinolysis, involving aPL-mediated activation of platelets, monocytes and endothelial cells, altered adhesion molecules and activation of pro-inflammatory cytokines, complement and neutrophil activation, and tissue factor upregulation and many others (3, 25, 26). However, the correlation between antibody titers and different disease phenotypes and disease severity, as well as the progression from asymptomatic patients to catastrophic anti-phospholipid syndrome (CAPS), still leaves a gap in research, so scholars have tried to investigate the nature of APS disease from a new perspective at the genetic level.

A number of studies first analyzed the genomic diversity of APS patients. Barinotti A et al. found two siblings with APS and carried out whole exome sequencing to analyze single nucleotide polymorphisms (SNPS), and found that there were many sites involved in hemostasis, immune response and protein coding of phospholipid metabolism. However, the correlation with APS is weak (27). In Meta-analysis, Islam et al. showed that 16 genes related to thrombolic APS (TAPS), and the proteins encoded by these genes were interrelated, mainly involved in two processes of coagulation cascade and immune response (28).

Some studies have also proposed that the expression of type-I interferons (IFN-1) gene is related to the pathogenesis of APS and other autoimmune diseases (29). In 2008, the results of a genome-wide array study showed that a total of 93 genes were differentially expressed between primary APS patients and healthy controls, with both type I and type II IFN-induced families showing upregulated expression in PAPS subjects (30). Perez-Sanchez et al. analyzed monocyte gene expression and found the similar differences in interferon signature genes in patients with PAPS and APS associated with SLE (31). Further transcriptome sequencing of neutrophils from primary APS patients and healthy controls revealed that IFIT1 (IFN-1 regulatory gene) was the most up-regulated gene, and IFIT3, IFI6, MX1 and HERC5 were also significantly increased in APS (32). These findings also suggest that changes in IFN gene expression may lead to activation of multiple pathogenic signaling pathways in APS.

In addition, complement system activation also plays a key role in aPLs-mediated pregnancy loss (33, 34). In the study of Chaturvedi et al, DNA exome capture sequencing was performed on CAPS patient samples, including 15 exons related to complement activation and regulation. The results showed that CAPS patients had a high rare germline variation rate (60%) in complement regulatory genes. The mutation rate (51.5%) was similar to that of atypical heomlytic uremic syndrome patients (35). However, this is still a controversial issue, as researchers have found high circulating levels of complement fission products in asymptomatic aPLs carriers (36). Samantha L Coss et al. (37) discuss the complex genetic polymorphisms in complement and their association with autoimmune diseases, potential mutations in these genes act as a strongly predisposing factor, leading to uncontrolled complement activation and a more severe thrombotic phenotype. Clearly, hemostasis and complement activation feature in the induction of thrombosis by aPLs. Since these enzymatic cascades are intrinsically linked, coagulation activation may be the cause and subsequent complement activation may be the consequence.

Recently, in a large whole-exome sequencing study of ethnic populations, variants with low allele counts were filtered and analyzed in combination with SNP frequency, amino acid substitution tolerance and mutation-carrying domain function, which revealed rare variants of P2RY8 allele were found in SLE and APS patients. Further investigation of the effect of P2RY8 variants on protein function indicates that the enhancement of P2RY8 signaling pathway may have the potential to prevent or treat systemic autoimmune diseases (38). The current diagnostic criteria for systemic autoimmune diseases are often ambiguous, ununiversal, or difficult to evaluate, which can lead to misdiagnosis or leave patients undiagnosed. However, there are widespread clinical and genetic similarities between rheumatic autoimmune diseases (39). The PRECISESADS project now has begun to reclassify major systemic autoimmune diseases based on underlying molecular mechanisms (40, 41).

These overlapping and complementary findings also prove that the clinical manifestations and disease course of APS are highly heterogeneous with many different pathogenesis. There are still many challenges in the study of its genetic background. However, the sequencing technology can further discover a variety of possible changes in the gene level of this disease, which is expected to integrate and verify various research data, discover new potential diagnostic markers and therapeutic targets in in-depth studies, provide evidence support for the development of detailed and personalized treatment strategies.

## 3 Mining novel diagnostic biomarkers for APS based on high-throughput sequencing technology

### 3.1 Differential diagnosis of APS from other autoimmune diseases

Autoimmune diseases are a large category of diseases that may cause tissue damage due to the body’s immune response to autoantigens. The clinical symptoms of patients with all kinds of diseases are relatively similar, and patients suffering from other autoimmune diseases are also likely to have abnormal elevated aPLs. For example, about 30%-40% patients with SLE can be detected as positive standard aPLs. The antibody titer is strongly correlated with the incidence of SLE complications (42). This will cause confusion in clinical differential diagnosis to some extent. If a good distinction can be made in the diagnosis and treatment process, it will provide great benefits for precision medicine. Although there have been studies on the diagnostic value of non-standard aPL in APS (1, 43–47), most of them have a small number of cases and a large difference in the positive rate of aPLs testing, which cannot be truly translated into clinical application.

As early as 2009, Hong Yin et al. (48, 49) found that *STAT4* and *BLK* had strong genetic correlation with primary APS. Also *IRF5* is associated with primary APS, but is not a major risk factor. Despite clinical and serological similarities between SLE and primary APS, there is a strong genetic association with a few SLE susceptibility genes and no more complex genetic association, which may result in a lack of full-blown SLE in patients with primary APS. Perez-Sanchez et al. (50) further analyzed the gene expression profiles in peripheral blood of primary APS patients, SLE patients with secondary APS, SLE patients and healthy controls, and found that there were significant differences in gene expression among all the groups, among which 20-30% of the differentially expressed genes were associated with atherosclerosis, inflammation and cardiovascular diseases. There is a complex network of shared genetic loci in immune diseases, in which 69% of the loci associated with each disease in autoimmune diseases are shared with other types of autoimmune diseases. This suggests that there are many shared pathways among autoimmune diseases (39, 51). These researchers combined genomic data with clinical evidence, and transcriptome and protein mapping profiles are added to identify related genes, microRNAs and many signaling pathways of monocyte subsets that may promote APS development (52), which providing directions for deeper research in the next stage. However, genomic analysis does not provide sufficient resolution to explain the complex pathological features of patients with autoimmune diseases, and epigenetic modifications may be closely related to immune responses as additional modulators. In the latest study, researchers (53) analyzed the genome-wide DNA methylation characteristics of neutrophils in APS patients. After multiple rounds of careful verification, it was determined that the single probe hypomethylation of IFI44L promoter (Cg06872964) had the highest accuracy in distinguishing SLE and APS patients, with a sensitivity and specificity of 93.3% and 80.0%, respectively. It has good differential diagnosis ability and is expected to be used in clinical translation after multi-center cohort validation. Interestingly, an association was noted between differential methylation in PAPS and genetic regions that regulate pregnancy, such as ETS1 and EMP2. In the future, with the combination of deep sequencing data mining and multi-omics map changes, more APS biomarkers with higher specificity can be screened out. Combined with the antibody titer information, it will provide corresponding reference for the auxiliary differential diagnosis of APS and other self-immune diseases.

### 3.2 Differential diagnosis of primary APS and secondary APS

Primary APS refers to those that meet the diagnostic criteria of APS and have no other autoimmune diseases or other diseases induced by aPL, such as infection, malignant tumors, and hemodialysis drugs. Secondary APS refers to APS secondary to other diseases resulting in related clinical symptoms or manifestations. For patients with secondary APS, most of them are secondary to SLE, primary immunologic thrombocytopenic purpura (ITP) and other autoimmune diseases (54). In the actual clinical diagnosis and treatment of APS patients, primary and secondary disease cannot be distinguished based on the results of other biomarkers such as aPL. At present, it can only be determined by the patient’s detailed history of concomitant other autoimmune diseases and the relationship between them. Although the Sydney Standard does not mention a strict distinction between primary and secondary APS, EULAR makes relevant recommendations for the management of APS patients (55). It is also crucial to determine which is the initiator of clinical adverse events in clinical intervention. In the pediatric population, the distribution of PAPS and SAPS tends to be similar (56). It has also been suggested that children may progress from primary APS to secondary APS more often (57). Some studies have shown that there are differences between the two (58). After comparing the HLA-DRB1 and HLA-DQB1 profiles of primary APS and secondary APS patients, it was found that the frequency of DRB1*13, DQB1*06 and DQB1*0302 alleles was high in primary APS patients, while the DQB1*0301 allele was rare (59). In addition, primary APS patients and secondary APS patients show different characteristics in mitochondrial function, oxidative stress, interferon signaling, and various genes mediating atherosclerosis and inflammatory signaling, suggesting that they may have different pathways in the development of the disease (60). However, repeated studies with large samples and different ethnic groups are needed to further explore the underlying differences.

### 3.3 Differential diagnosis of APS subtypes

According to the different clinical manifestations of APS patients, APS can be divided into several subtypes. The subgroup of APS, obstetric antiphospholipid syndrome (OAPS), is defined as persistent positivity for aPL and early recurrent pregnancy loss, early fetal death, stillbirth, or preterm delivery in the first 34 weeks of gestation due to preeclampsia, eclampsia, and placental insufficiency (61). Thus, OAPS is considered as the most common acquired risk factor for treatable causes of recurrent pregnancy loss and represents a significant health burden for women of childbearing age. Thrombosis is the most common manifestation of APS and is usually the first symptom in thrombotic antiphospholipid syndrome (TAPS) patients, including venous thromboembolism, arterial thrombosis and microvascular thrombosis, which impairs the peripheral vasculature, cerebral vasculature and the vasculature of the heart, lungs, kidneys and other organs (62, 63). CAPS refers to the widespread thrombosis of small blood vessels or large blood vessels in the whole body in a few patients within a short period of time, usually within a few days to a few weeks, causing ischemia and necrosis of multiple organs, also known as severe antiphospholipid syndrome (64). Current diagnostic criteria for OAPS include unexplained recurrent abortion (at least 3 consecutive times and more than 10 weeks), at least 1 fetal death (more than 10 weeks) or premature (less than 34 weeks). This standard has remained unchanged since its formulation in 2006 (54). In practical clinical application, patients with other types of clinical pregnancy adverse events and suspected APS cannot be diagnosed. Vascular and obstetric APS share some similarities, but can also be different, making APS still a complex clinical disorder. Although thrombosis is the primary clinical manifestation of vascular APS, non-thrombotic mechanisms are considered more important than placental infarction in the pathogenesis of obstetric APS (65). Therefore, the OAPS Working group emphasized the need for new OAPS standards at the 16th International Antiphospholipid Antibody Congress (66) and the work has made some progress (13).

A genome-wide asociation study in a Japanese population with OAPS showed that the specific genotype of thyroid stimulating hormone receptor (TSHR) gene and C1D nuclear receptor corepressor gene (C1D) may be a risk factor for OAPS (67). If the new research results can be included in the new standards in the future, it is expected to supplement the application of NGS in prenatal diagnosis, which may improve the accessibility of prenatal diagnosis to a certain extent and alleviate the current clinical pressure.

For the diagnosis of TAPS, Yomna K et al. found that protein Z-79 G/A gene polymorphism could prevent APS thrombosis (68). Plasin Rodriguez et al. also tested PROCR, the gene encoding endothelial cell protein C receptor. This indicates that PROCR H1 haploid type may have a protective effect on APS thrombosis (69). Also in a Chinese study, the C677T mutation of MTHFR was found to be a risk factor for arterial thrombosis in Chinese Han patients with APS (70). In addition, aβ2GPI-β2GPI complex is closely related to the pathogenesis of APS. Some studies have proposed that increased nitrated β2GPI may affect platelet function in APS patients, and elevated nitrated β2GPI level is a potential indicator of increased risk of thrombosis (71), which has the opportunity to be used to characterize TAPS disease status in the future.

For the discrimination between different APS subtypes, Perez-Sanchez (72) analyzed the characteristics of miRNAs expression profiles in blood samples of 90 APS patients, among which 39 differentially expressed miRNAs sets could be used as disease biomarkers. Not only was this collection able to accurately identify patients with APS in the study population, different subsets were also associated with adverse pregnancy events and thrombosis. If the relevant diagnostic model is established using artificial intelligence algorithm, TAPS and OAPS can be crearly distinguished. Some scholars have developed a new way to study other sample types of APS patients. The clinical collection of urine samples is more convenient and less painful for patients than blood samples. Zhou Z et al. tried to start with the urine samples of APS patients, conducted genomics and proteomics tests at the same time. They found that TAPS and OAPS are distributed with different urinary protein patterns. The level of C-X-C Motif Chemokine Ligand 12 (CXCL12) in the urine of TAPS patients was higher than that of OAPS patients. On the contrary, the level of platelet derived growth factor-β (PDGF-β) was lower than that of OAPS patients, suggesting that CXCL12 and PDGF-β in urine may be used as potential noninvasive markers for the identification of TAPS and OAPS (73). The current evidence is insufficient to recommend routine clinical testing of these novel markers, but the evaluation and confirmation of these markers are still under development. It is believed that such new markers with potential for rapid and automated detection will become the point of clinical transformation in the future through multi-dimensional verification by scholars.

## 4 Research exploration on prevention, prognosis and management of APS

The etiology of APS is not well understood, and there are no effective measures to prevent APS. For patients without thrombotic events, especially women of childbearing age, the guidelines recommend thst lifestyle changes to prevent adverse events (74).

For the prognosis and management of APS patients, Amigo et al. (75) have developed a system to assess aPL-related organ damage in patients with APS. Experts in this field have developed several tools to assess the risk of recurrent thrombosis or pregnancy morbidity in APS based on the diverse characteristics of the disease population (76–82). Recent studies have shown that JAK2^V617F^ mutation can lead to an increased risk of thrombolism. For APS patients with JAK2^V617F^ mutation, thrombotic adverse events may occur at an earlier stage, and more aggressive antithrombotic therapy strategies are needed to treat and prevent recurrence (83). Moreover, genetic prethrombotic risk factors are well established in children with APS, with 45% of patients enrolled in the Ped-APS registry having more than one genetic prethrombotic risk factors. Pediatric APS patients often present with “non-classical” APL-related clinical findings that cannot be directly attributed to the thrombotic process, implying a clear distinction between SLE related and APL-related manifestations (56). Through a large range of long-term follow-up observation data, the prediction accuracy can be improved if related indexes such as genetics and genes can be added to the relevant prediction algorithm, but more verification is still needed.

## 5 Discussion

In the past few years, many studies (**Table 1**) have been conducted on genetic polymorphisms in APS patients, which provide references for the future use of molecular mapping to characterize patients. The application of NGS technology will obtain relatively complete characteristics and information of APS patients, including specific immune cell populations, molecular cloning and gene expression differences, etc. The data system is huge and complex. Clarifying effective parameter information closely related to the diagnosis of APS, thrombosis and pathological pregnancy is an important step in screening potential biomarkers specific for APS. Compared with clinical antibody detection, NGS method can greatly improve the sensitivity and specificity of detection. By increasing the sequencing depth, a large volume of differential information can be obtained, but at the same time, the detection cost is correspondingly increased. Therefore, for the application of NGS in the current post-clinical practice, relevant examination at the gene level should be mainly evaluated and validated in the APS population with certain thrombotic and obstetric manifestations, maybe gradually conducted for patients with no or low expression of aPLs antibody during the preliminary screening, but highly suspected of APS, to assist in early diagnosis and supplement the problem that APS diagnostic system dominated by standard aPLs cannot meet the needs of clinical diagnosis and treatment in the future.

**Table 1:** Recent studies on genetic polymorphism in patients with APS.

genetic polymorphism	conclusion	cite
TSHR and C1D	demonstrate that a specific genotype of TSHR and C1D genes can be a risk factor for obstetric APS.	(67)
Z-79 G/A	protein Z-79 G/A gene polymorphism may have a protective value against thrombosis in APS.	(68)
PROCR	PROCR H1 haplotype was less frequently found in APS patients with arterial thrombosis, suggesting a protective effect of PROCR H1 against arterial thrombosis in these patients.	(69)
MTHFR C677T	the C677T mutation of MTHFR is a risk factor for arterial thrombosis in Chinese Han patients with APS.	(70)
JAK2^V617F^	APS patients carrying the JAK2V617F mutation may lead to earlier manifestation of thromboembolic events.	(83)
NCF1-339	reveal a striking connection between the reactive oxygen species deficient NCF1-339 genotypes and the presence of APS.	(84)

It is expected that in the future, through clinical studies with a relatively large sample size, strict patient inclusion criteria will be set up. APS patients will be stratified according to clinical outcomes and aPLs expression profiles of patients, so as to obtain experimental data clearly related to APS clinical manifestations and aPLs expression while reducing the occurrence of analysis bias. We need to ensure the independence of the derived data as biomarkers, analyze the causes of various differences, enrich the pathogenesis of APS, and further confirm its value in the diagnosis and prognosis of APS. Further work should be focused on building on existing work to evaluate genetic risk factors that significantly contribute to thrombosis and adverse pregnancy events through multilevel and multi-dimensional bioinformatics analysis, and to better identify standard and non-standard clinical phenotypes, antiphospholipid antibody profiles, and their relevance to clinical outcomes, prognostic tools, and treatment strategies. Sequencing information can be combined with multiomics techniques, opening the door to the possibility of developing novel diagnostic criteria for APS and providing more options for targeted therapies to prevent clinical adverse events in APS.

## Author contributions

QL designed and wrote the review, drew the tables. YT and SY wrote and revised this manuscript. LC revised this manuscript and reviewed the figures and tables. All authors gave final approval for publication.

## References

[1] Iwaniec T Kaczor MP Celińska-Löwenhoff M Polański S Musiał J. Clinical significance of anti-domain 1 β2-glycoprotein I antibodies in antiphospholipid syndrome. Thromb Res (2017) 153:90–4. doi: 10.1016/j.thromres.2017.02.019 28363116

[2] Rodriguez-Pintó I Espinosa G Cervera R. Precision medicine and the antiphospholipid syndrome: what is the future? Clin Rheumatol (2020) 39(4):1015–7. doi: 10.1007/s10067-020-04987-8 32060811

[3] Ruiz-Irastorza G Crowther M Branch W Khamashta MA. Antiphospholipidsyndrome Lancet (2010) 376(9751):1498–509. doi: 10.1016/S0140-6736(10)60709-X 20822807

[4] Cervera R. Antiphospholipid syndromes. Thromb Res (2017) 151 Suppl 1:S43–7. doi: 10.1016/S0049-3848(17)30066-X 28262233

[5] Huang S Ninivaggi M Chayoua W de Laat BVWF. Platelets and the antiphospholipid syndrome. Int J Mol Sci (2021) 22(8):4200. doi: 10.3390/ijms22084200 33919627 PMC8074042

[6] Leal Rato M Bandeira M Romão VC Aguiar de Sousa D. Neurologic manifestations of the antiphospholipid syndrome - an update. Curr Neurol Neurosci Rep (2021) 21(8):41. doi: 10.1007/s11910-021-01124-z 34125304 PMC8200381

[7] Rosina S Chighizola CB Ravelli A Cimaz R. Pediatric antiphospholipid syndrome: from pathogenesis to clinical management. Curr Rheumatol Rep (2021) 23(2):10. doi: 10.1007/s11926-020-00976-7 33511497 PMC7843475

[8] El Hasbani G Taher AT Sciascia S Uthman I. Antiphospholipid syndrome: the need for new international classification criteria. Expert Rev Clin Immunol (2021) 17(4):385–94. doi: 10.1080/1744666X.2021.1900733 33682558

[9] Hughes GR Khamashta MA. Seronegative antiphospholipid syndrome. Ann Rheum Dis (2003) 62(12):1127. doi: 10.1136/ard.2003.006163 14644846 PMC1754381

[10] Pires da Rosa G Bettencourt P Rodríguez-Pintó I Cervera R Espinosa G. "Non-criteria" antiphospholipid syndrome: a nomenclature proposal. Autoimmun Rev (2020) 19(12):102689. doi: 10.1016/j.autrev.2020.102689 33223008

[11] Chighizola CB Pregnolato F Andreoli L Bodio C Cesana L Comerio C. Beyond thrombosis: anti-β2GPI domain 1 antibodies identify late pregnancy morbidity in anti-phospholipid syndrome. J Autoimmun (2018) 90:76–83. doi: 10.1016/j.jaut.2018.02.002 29454510

[12] Liu X Zhu L Liu H Cai Q Yun Z Sun F. Non-criteria antiphospholipid antibodies in antiphospholipid syndrome: diagnostic value added. Front Immunol (2022) 13:972012. doi: 10.3389/fimmu.2022.972012 36389827 PMC9643638

[13] Barbhaiya M Zuily S Ahmadzadeh Y Amigo MC Avcin T Bertolaccini ML. Development of a new international antiphospholipid syndrome classification criteria phase I/II report: generation and reduction of candidate criteria. Arthritis Care Res (2021) 73(10):1490–150. Hoboken. doi: 10.1002/acr.24520 PMC8966711 33253499

[14] Cervera R Rodríguez-Pintó I Legault K Erkan D. 16th international congress on antiphospholipid antibodies task force report on catastrophic antiphospholipid syndrome. Lupus (2020) 29(12):1594–600. doi: 10.1177/0961203320951260 32819183

[15] Djokovic A Stojanovich L. Summary of the 12th Meeting of the European Forum on antiphospholipid antibodies ONLINE. Lupus (2021) 30(13):2162–4. doi: 10.1177/09612033211055010 34696636

[16] Petri M. Epidemiology of the antiphospholipid antibody syndrome. J Autoimmun (2000) 15(2):145–51. doi: 10.1006/jaut.2000.0409 10968901

[17] Vikerfors A Johansson AB Gustafsson JT Jönsen A Leonard D Zickert A. Clinical manifestations and anti-phospholipid antibodies in 712 patients with systemic lupus erythematosus: evaluation of two diagnostic assays. Rheumatol (Oxford) (2013) 52(3):501–9. doi: 10.1093/rheumatology/kes252 23159889

[18] Yohe S Thyagarajan B. Review of clinical next-generation sequencing. Arch Pathol Lab Med (2017) 141(11):1544–57. doi: 10.5858/arpa.2016-0501-RA 28782984

[19] Koboldt DC Steinberg KM Larson DE Wilson RK Mardis ER. The next-generation sequencing revolution and its impact on genomics. Cell (2013) 155(1):27–38. doi: 10.1016/j.cell.2013.09.006 24074859 PMC3969849

[20] Bao S Jiang R Kwan W Wang B Ma X Song YQ. Evaluation of next-generation sequencing software in mapping and assembly. J Hum Genet (2011) 56(6):406–14. doi: 10.1038/jhg.2011.43 21525877

[21] van Zeggeren IE Edridge AWD van de Beek D Deijs M Koekkoek SM Wolthers KC. Diagnostic accuracy of VIDISCA-NGS in patients with suspected central nervous system infections. Clin Microbiol Infect (2021) 27(4):631.e7–631.e12. doi: 10.1016/j.cmi.2020.06.012 32590059

[22] Astur N Maciel BFB Doi AM Martino MDV Basqueira MS Wajchenberg M. Next-generation sequencing (NGS) to determine microbiome of herniated intervertebral disc. Spine J (2022) 22(3):389–98. doi: 10.1016/j.spinee.2021.09.005 34547388

[23] Rehder C Bean LJH Bick D Chao E Chung W Das S. Next-generation sequencing for constitutional variants in the clinical laboratory, 2021 revision: a technical standard of the American college of medical genetics and genomics (ACMG). Genet Med (2021) 23(8):1399–415. doi: 10.1038/s41436-021-01139-4 33927380

[24] Erkan D Sciascia S Bertolaccini ML Cohen H APS ACTION Executive Committee. Antiphospholipid syndrome alliance for clinical trials and international networking (APS ACTION): 10-year update. Curr Rheumatol Rep (2021) 23(6):45. doi: 10.1007/s11926-021-01008-8 33932165 PMC8088198

[25] Gandhi AA Estes SK Rysenga CE Knight JS. Understanding the pathophysiology of thrombotic APS through animal models. Int J Mol Sci (2021) 22(5):2588. doi: 10.3390/ijms22052588 33806694 PMC7961365

[26] Green D. Pathophysiology of antiphospholipid syndrome. Thromb Haemost (2022) 122(7):1085–95. doi: 10.1055/a-1701-2809 34794200 PMC9391091

[27] Barinotti A Radin M Cecchi I Foddai SG Rubini E Roccatello D. Genetic factors in antiphospholipid syndrome: preliminary experience with whole exome sequencing. Int J Mol Sci (2020) 21(24):9551. doi: 10.3390/ijms21249551 33333988 PMC7765384

[28] Islam MA Khandker SS Alam F Kamal MA Gan SH. Genetic risk factors in thrombotic primary antiphospholipid syndrome: a systematic review with bioinformatic analyses. Autoimmun Rev (2018) 17(3):226–43. doi: 10.1016/j.autrev.2017.10.014 29355608

[29] Cecchi I Radin M Rodríguez-Carrio J Tambralli A Knight JS Sciascia S. Utilizing type I interferon expression in the identification of antiphospholipid syndrome subsets. Expert Rev Clin Immunol (2021) 17(4):395–406. doi: 10.1080/1744666X.2021.1901581 33686921 PMC10183148

[30] Bernales I Fullaondo A Marín-Vidalled MJ Ucar E Martínez-Taboada V López-Hoyos M. Innate immune response gene expression profiles characterize primary antiphospholipid syndrome. Genes Immun (2008) 9(1):38–46. doi: 10.1038/sj.gene.6364443 17960154

[31] Perez-Sanchez C Barbarroja N Messineo S Ruiz-Limon P Rodriguez-Ariza A Jimenez-Gomez Y. Gene profiling reveals specific molecular pathways in the pathogenesis of atherosclerosis and cardiovascular disease in antiphospholipid syndrome, systemic lupus erythematosus and antiphospholipid syndrome with lupus. Ann Rheum Dis (2015) 74(7):1441–9. doi: 10.1136/annrheumdis-2013-204600 24618261

[32] Knight JS Meng H Coit P Yalavarthi S Sule G Gandhi AA. Activated signature of antiphospholipid syndrome neutrophils reveals potential therapeutic target. JCI Insight (2017) 2(18):e93897. doi: 10.1172/jci.insight.93897 28931754 PMC5621930

[33] Kim MY Guerra MM Kaplowitz E Laskin CA Petri M Branch DW. Complement activation predicts adverse pregnancy outcome in patients with systemic lupus erythematosus and/or antiphospholipid antibodies. Ann Rheum Dis (2018) 77(4):549–55. doi: 10.1136/annrheumdis-2017-212224 PMC6037302 29371202

[34] Chaturvedi S Braunstein EM Brodsky RA. Antiphospholipid syndrome: complement activation, complement gene mutations, and therapeutic implications. J Thromb Haemost (2021) 19(3):607–16. doi: 10.1111/jth.15082 PMC8080439 32881236

[35] Chaturvedi S Braunstein EM Yuan X Yu J Alexander A Chen H. Complement activity and complement regulatory gene mutations are associated with thrombosis in APS and CAPS. Blood (2020) 135(4):239–51. doi: 10.1182/blood.2019003863 PMC6978159 31812994

[36] Lonati PA Scavone M Gerosa M Borghi MO Pregnolato F Curreli D. Blood cell-bound C4d as a marker of complement activation in patients with the antiphospholipid syndrome. Front Immunol (2019) 10:773. doi: 10.3389/fimmu.2019.00773 31031764 PMC6474283

[37] Coss SL Zhou D Chua GT Aziz RA Hoffman RP Wu YL. The complement system and human autoimmune diseases. J Autoimmun (2022) 17:102979. doi: 10.1016/j.jaut.2022.102979 PMC10276174 36535812

[38] He Y Gallman AE Xie C Shen Q Ma J Wolfreys FD. P2RY8 variants in lupus patients uncover a role for the receptor in immunological tolerance. J Exp Med (2022) 219(1):e20211004. doi: 10.1084/jem.20211004 34889940 PMC8669517

[39] Barturen G Beretta L Cervera R Van Vollenhoven R Alarcón-Riquelme ME. Moving towards a molecular taxonomy of autoimmune rheumatic diseases. Nat Rev Rheumatol (2018) 14(2):75–93. doi: 10.1038/nrrheum.2017.220 29362467

[40] Barturen G Babaei S Català-Moll F Martínez-Bueno M Makowska Z Martorell-Marugán J. Integrative analysis reveals a molecular stratification of systemic autoimmune diseases. Arthritis Rheumatol (2021) 73(6):1073–85. doi: 10.1002/art.41610 33497037

[41] Simon Q Grasseau A Boudigou M Le Pottier L Bettachioli E Cornec D. A proinflammatory cytokine network profile in Th1/Type 1 effector b cells delineates a common group of patients in four systemic autoimmune diseases. Arthritis Rheumatol (2021) 73(8):1550–61. doi: 10.1002/art.41697 33605069

[42] Ünlü O Zuily S Erkan D. The clinical significance of antiphospholipid antibodies in systemic lupus erythematosus. Eur J Rheumatol (2016) 3(2):75–84. doi: 10.5152/eurjrheum.2015.0085 27708976 PMC5042235

[43] Shi H Zheng H Yin YF Hu QY Teng JL Sun Y. Antiphosphatidylserine/prothrombin antibodies (aPS/PT) as potential diagnostic markers and risk predictors of venous thrombosis and obstetric complications in antiphospholipid syndrome. Clin Chem Lab Med (2018) 56(4):614–24. doi: 10.1515/cclm-2017-0502 29166262

[44] Ortona E Capozzi A Colasanti T Conti F Alessandri C Longo A. Vimentin/cardiolipin complex as a new antigenic target of the antiphospholipid syndrome. Blood (2010) 116(16):2960–7. doi: 10.1182/blood-2010-04-279208 20634382

[45] Litvinova E Darnige L Kirilovsky A Burnel Y de Luna G Dragon-Durey MA. Prevalence and significance of non-conventional antiphospholipid antibodies in patients with clinical APS criteria. Front Immunol (2018) 9:2971. doi: 10.3389/fimmu.2018.02971 30619328 PMC6302212

[46] Zhang S Wu Z Zhang W Zhang F Li Y Liu Y. Clinical performance of non-criteria antibodies to phospholipids in Chinese patients with antiphospholipid syndrome. Clin Chim Acta (2019) 495:205–9. doi: 10.1016/j.cca.2019.04.065 31002781

[47] Mekinian A Bourrienne MC Carbillon L Benbara A Noémie A Chollet-Martin S. Non-conventional antiphospholipid antibodies in patients with clinical obstetrical APS: prevalence and treatment efficacy in pregnancies. Semin Arthritis Rheum (2016) 46(2):232–7. doi: 10.1016/j.semarthrit.2016.05.006 27432776

[48] Fredi M Tincani A Yin H Delgado-Vega AM Borghi MO Meroni PL. IRF5 is associated with primary antiphospholipid syndrome, but is not a major risk factor. Arthritis Rheum (2010) 62(4):1201–2. doi: 10.1002/art.27345 20131273

[49] Yin H Borghi MO Delgado-Vega AM Tincani A Meroni PL Alarcón-Riquelme ME. Association of STAT4 and BLK, but not BANK1 or IRF5, with primary antiphospholipid syndrome. Arthritis Rheum (2009) 60(8):2468–71. doi: 10.1002/art.24701 19644876

[50] Pérez-Sánchez C Aguirre MA Ruiz-Limón P Barbarroja N Jiménez-Gómez Y de la Rosa IA. 'Atherothrombosis-associated microRNAs in antiphospholipid syndrome and systemic lupus erythematosus patients'. Sci Rep (2016) 6:31375. doi: 10.1038/srep31375 27502756 PMC4977549

[51] Farh KK Marson A Zhu J Kleinewietfeld M Housley WJ Beik S. Genetic and epigenetic fine mapping of causal autoimmune disease variants. Nature (2015) 518(7539):337–43. doi: 10.1038/nature13835 PMC4336207 25363779

[52] Pérez-Sánchez L Patiño-Trives AM Aguirre-Zamorano MÁ Luque-Tévar M Ábalos-Aguilera MC Arias-de la Rosa I. Characterization of antiphospholipid syndrome atherothrombotic risk by unsupervised integrated transcriptomic analyses. Arterioscler Thromb Vasc Biol (2021) 41(2):865–77. doi: 10.1161/ATVBAHA.120.315346 33356391

[53] Weeding E Coit P Yalavarthi S Kaplan MJ Knight JS Sawalha AH. Genome-wide DNA methylation analysis in primary antiphospholipid syndrome neutrophils. Clin Immunol (2018) 196:110–6. doi: 10.1016/j.clim.2018.11.011 PMC6413498 30471352

[54] Miyakis S Lockshin MD Atsumi T Branch DW Brey RL Cervera R. International consensus statement on an update of the classification criteria for definite antiphospholipid syndrome (APS). J Thromb Haemost (2006) 4(2):295–306. doi: 10.1111/j.1538-7836.2006.01753.x 16420554

[55] Tektonidou MG Andreoli L Limper M Amoura Z Cervera R Costedoat-Chalumeau N. EULAR recommendations for the management of antiphospholipid syndrome in adults. Ann Rheum Dis (2019) 78(10):1296–304. doi: 10.1136/annrheumdis-2019-215213 PMC11034817 31092409

[56] Avcin T Cimaz R Silverman ED Cervera R Gattorno M Garay S. Pediatric antiphospholipid syndrome: clinical and immunologic features of 121 patients in an international registry. Pediatrics (2008) 122(5):e1100–7. doi: 10.1542/peds.2008-1209 18955411

[57] Madison JA Zuo Y Knight JS. Pediatric antiphospholipid syndrome. Eur J Rheumatol (2019) 7(Suppl 1):1–10. doi: 10.5152/eurjrheum.2019.19160 PMC7004270 31804173

[58] Sangli SS Ryu JH Baqir M. Diffuse alveolar hemorrhage in primary versus secondary antiphospholipid syndrome. J Clin Rheumatol (2021) 27(8):e297–301. doi: 10.1097/RHU.0000000000001358 32195850

[59] Kapitany A Tarr T Gyetvai A Szodoray P Tumpek J Poor G. Human leukocyte antigen-DRB1 and -DQB1 genotyping in lupus patients with and without antiphospholipid syndrome. Ann N Y Acad Sci (2009) 1173:545–51. doi: 10.1111/j.1749-6632.2009.04642.x 19758197

[60] Belizna C Stojanovich L Cohen-Tervaert JW Fassot C Henrion D Loufrani L. Primary antiphospholipid syndrome and antiphospholipid syndrome associated to systemic lupus: are they different entities? Autoimmun Rev (2018) 17(8):739–45. doi: 10.1016/j.autrev.2018.01.027 29885541

[61] Schreiber K Sciascia S de Groot PG Devreese K Jacobsen S Ruiz-Izastorza G. Antiphospholipid syndrome. Nat Rev Dis Primers (2018) 4:17103. doi: 10.1038/nrdp.2017.103 29321641

[62] Cervera R Piette JC Font J Khamashta MA Shoenfeld Y Camps MT. Antiphospholipid syndrome: clinical and immunologic manifestations and patterns of disease expression in a cohort of 1,000 patients. Arthritis Rheum (2002) 46(4):1019–27. doi: 10.1002/art.10187 11953980

[63] Cervera R Serrano R Pons-Estel GJ Ceberio-Hualde L Shoenfeld Y de Ramón E. Morbidity and mortality in the antiphospholipid syndrome during a 10-year period: a multicentre prospective study of 1000 patients. Ann Rheum Dis (2015) 74(6):1011–8. doi: 10.1136/annrheumdis-2013-204838 24464962

[64] Garcia D Erkan D. Diagnosis and management of the antiphospholipid syndrome. N Engl J Med (2018) 378(21):2010–21. doi: 10.1056/NEJMra1705454 29791828

[65] Meroni PL Borghi MO Grossi C Chighizola CB Durigutto P Tedesco F. Obstetric and vascular antiphospholipid syndrome: same antibodies but different diseases? Nat Rev Rheumatol (2018) 14(7):433–40. doi: 10.1038/s41584-018-0032-6 29891914

[66] de Jesús GR Benson AE Chighizola CB Sciascia S Branch DW. 16th international congress on antiphospholipid antibodies task force report on obstetric antiphospholipid syndrome. Lupus (2020) 29(12):1601–15. doi: 10.1177/0961203320954520 32883160

[67] Sugiura-Ogasawara M Omae Y Kawashima M Toyo-Oka L Khor SS Sawai H. The first genome-wide association study identifying new susceptibility loci for obstetric antiphospholipid syndrome. J Hum Genet (2017) 62(9):831–8. doi: 10.1038/jhg.2017.46 28424481

[68] Eissa YK Ellithy HN Yousry SM Ismail Z. The relation between protein z polymorphism and the risk of thrombosis in Egyptian patients with antiphospholipid syndrome. Hematol Oncol Stem Cell Ther (2018) 11(4):219–24. doi: 10.1016/j.hemonc.2018.03.005 29684342

[69] Plasín-Rodríguez MA Rodríguez-Pintó I Patricio P Monteagudo J Cervera R Reverter JC. The H1 haplotype of the endothelial protein c receptor protects against arterial thrombosis in patients with antiphospholipid syndrome. Thromb Res (2018) 169:128–34. doi: 10.1016/j.thromres.2018.07.006 30048851

[70] Tang Z Shi H Liu H Cheng X Su Y Ye J. Methylenetetrahydrofolate reductase 677T allele is a risk factor for arterial thrombosis in Chinese han patients with antiphospholipid syndrome. Biomedicines (2022) 11(1):55. doi: 10.3390/biomedicines11010055 36672563 PMC9856080

[71] Krilis M Qi M Ioannou Y Zhang JY Ahmadi Z Wong JWH. Clinical relevance of nitrated beta 2-glycoprotein I in antiphospholipid syndrome: implications for thrombosis risk. J Autoimmun (2021) 122:102675. doi: 10.1016/j.jaut.2021.102675 34098405

[72] Pérez-Sánchez C Arias-de la Rosa I Aguirre MÁ Luque-Tévar M Ruiz-Limón P Barbarroja N. Circulating microRNAs as biomarkers of disease and typification of the atherothrombotic status in antiphospholipid syndrome. Haematologica (2018) 103(5):908–18. doi: 10.3324/haematol.2017.184416 PMC5927979 29545345

[73] Zhou Z You Y Wang F Sun Y Teng J Liu H. Urine proteomics differentiate primary thrombotic antiphospholipid syndrome from obstetric antiphospholipid syndrome. Front Immunol (2021) 12:702425. doi: 10.3389/fimmu.2021.702425 34489952 PMC8416615

[74] ESHRE Guideline Group on RPL Bender Atik R Christiansen OB Elson J Kolte AM Lewis S. ESHRE guideline: recurrent pregnancy loss. Hum Reprod Open (2018) 2:hoy004. doi: 10.1093/hropen/hoy004 PMC6276652 31486805

[75] Amigo MC Khamashta MA Cardiel M. Development and validation of an antiphospholipid damage index: a multicenter, binational project. Lupus (1998) 7(Suppl 1):68.

[76] Sciascia S Sanna G Murru V Roccatello D Khamashta MA Bertolaccini ML. GAPSS: the global anti-phospholipid syndrome score. Rheumatol (Oxford) (2013) 52(8):1397–403. doi: 10.1093/rheumatology/kes388 23315788

[77] Radin M Sciascia S Erkan D Pengo V Tektonidou MG Ugarte A. The adjusted global antiphospholipid syndrome score (aGAPSS) and the risk of recurrent thrombosis: results from the APS ACTION cohort. Semin Arthritis Rheum (2019) 49(3):464–8. doi: 10.1016/j.semarthrit.2019.04.009 PMC7402528 31153708

[78] Udry S Peréz SM Belizna C Aranda F Esteve-Valverde E Wingeyer SP. Clinical and therapeutic value of the adjusted global antiphospholipid syndrome score in primary obstetric antiphospholipid syndrome. Lupus (2022) 31(3):354–62. doi: 10.1177/09612033221078223 35157809

[79] Amigo MC Goycochea-Robles MV Espinosa-Cuervo G Medina G Barragán-Garfias JA Vargas A. Development and initial validation of a damage index (DIAPS) in patients with thrombotic antiphospholipid syndrome (APS). Lupus (2015) 24(9):927–34. doi: 10.1177/0961203315576858 25767071

[80] Otomo K Atsumi T Amengual O Fujieda Y Kato M Oku K. Efficacy of the antiphospholipid score for the diagnosis of antiphospholipid syndrome and its predictive value for thrombotic events. Arthritis Rheum (2012) 64(2):504–12. doi: 10.1002/art.33340 21953404

[81] Sciascia S Cosseddu D Montaruli B Kuzenko A Bertero MT. Risk scale for the diagnosis of antiphospholipid syndrome. Ann Rheum Dis (2011) 70(8):1517–8. doi: 10.1136/ard.2010.145177 21402561

[82] Pregnolato F Gerosa M Raimondo MG Comerio C Bartoli F Lonati PA. EUREKA algorithm predicts obstetric risk and response to treatment in women with different subsets of anti-phospholipid antibodies. Rheumatol (Oxford) (2021) 60(3):1114–24. doi: 10.1093/rheumatology/keaa203 32441742

[83] Janjetovic S Beckmann L Holstein K Rolling C Thiele B Schafhausen P. Prevalence of definite antiphospholipid syndrome in carriers of the JAK2V617F mutation. Thromb Res (2021) 198:55–61. doi: 10.1016/j.thromres.2020.11.027 33290883

[84] Linge P Arve S Olsson LM Leonard D Sjöwall C Frodlund M. NCF1-339 polymorphism is associated with altered formation of neutrophil extracellular traps, high serum interferon activity and antiphospholipid syndrome in systemic lupus erythematosus. Ann Rheum Dis (2020) 79(2):254–61. doi: 10.1136/annrheumdis-2019-215820 31704719

